# Escitalopram—translating molecular properties into clinical benefit: reviewing the evidence in major depression

**DOI:** 10.1177/0269881109349835

**Published:** 2010-08

**Authors:** Brian Leonard, David Taylor

**Affiliations:** 1 Department of Pharmacology, National University of Ireland, Galway, Ireland.; 2 Department of Psychiatry and Psychotherapy, Ludwig Maximilians University, Munich, Germany.; 3 Division of Pharmaceutical Sciences, King’s College, London, UK.; 4 Maudsley Hospital, London, UK.

**Keywords:** antidepressant, depression, escitalopram, stereoisomerism

## Abstract

The majority of currently marketed drugs contain a mixture of enantiomers; however, recent evidence suggests that individual enantiomers can have pharmacological properties that differ importantly from enantiomer mixtures. Escitalopram, the *S-*enantiomer of citalopram, displays markedly different pharmacological activity to the *R*-enantiomer. This review aims to evaluate whether these differences confer any significant clinical advantage for escitalopram over either citalopram or other frequently used antidepressants. Searches were conducted using PubMed and EMBASE (up to January 2009). Abstracts of the retrieved studies were reviewed independently by both authors for inclusion. Only those studies relating to depression or major depressive disorder were included. The search identified over 250 citations, of which 21 studies and 18 pooled or meta-analyses studies were deemed suitable for inclusion. These studies reveal that escitalopram has some efficacy advantage over citalopram and paroxetine, but no consistent advantage over other selective serotonin reuptake inhibitors. Escitalopram has at least comparable efficacy to available serotonin-norepinephrine reuptake inhibitors, venlafaxine XR and duloxetine, and may offer some tolerability advantages over these agents. This review suggests that the mechanistic advantages of escitalopram over citalopram translate into clinical efficacy advantages. Escitalopram may have a favourable benefit-risk ratio compared with citalopram and possibly with several other antidepressant agents.

## Introduction

The selective serotonin reuptake inhibitor (SSRI) class of antidepressants has had a substantial impact on the treatment of depression since its introduction in the mid-1980s. Escitalopram is an SSRI that is the most selective serotonin-specific antidepressant marketed to date (Lam and Annemans, 2007). Escitalopram is the *S*-enantiomer of the racemate citalopram. The efficacy of citalopram as an antidepressant is almost entirely due to the activity of escitalopram; the *S*-enantiomer is approximately 150 times more potent as a reuptake inhibitor than *R*-citalopram in vitro (Hyttel et al., 1992). The mode of action of escitalopram also differs from *R*-citalopram in that it displays a unique interaction with the human serotonin reuptake transporter (SERT) and there is evidence to suggest that it has a self-potentiating effect on the SERT, which is mediated by an allosteric binding site that is distinct from the primary, high-affinity binding site on the SERT (Plenge et al., 2007).

There is a growing trend to develop drugs that comprise a single enantiomer, as opposed to a mixture of enantiomers. This trend has largely been prompted by the need to develop drugs with improved tolerability profiles, and it is supported by recommendations issued by the Food and Drug Administration (FDA) and the European Medicines Agency for the development of chiral drugs (Food and Drug Administration, 1992; Committee for Proprietary Medical Products, 1993). Although the racemic nature of drugs has been recognized for many years, it was frequently assumed that only one enantiomer was pharmacologically active; the other enantiomer was considered inactive, or was assumed merely to contribute to the side-effect profile of the drug (Ariens et al., 1983; Waldeck, 2003). However, extensive research has shown that two enantiomers can behave synergistically or antagonistically. Consequently, drugs that are racemates should be considered to be a mixture of potentially different drugs, with potentially different therapeutic and adverse properties (Cordato et al., 2003; McConathy and Owens, 2003); for example, the β-blocker labetalol comprises four different stereoisomer combinations, which can have markedly different physiological effects (Brittain et al., 1982). Single enantiomer drugs, such as escitalopram, can, therefore, potentially offer a number of distinct benefits, specifically with respect to inter-patient pharmacokinetic and pharmacodynamic variability, as well as reducing toxicity arising from the presence of the therapeutically redundant enantiomers (Leonard, 2001) and improving therapeutic efficacy.

In Europe, escitalopram is currently approved for use in major depressive disorder (MDD), panic disorder, social anxiety disorder, generalized anxiety disorder and obsessive compulsive disorder (Baldwin et al., 2007). Prior to approval of escitalopram in 2002 for the treatment of MDD in the USA, the FDA conducted an extensive review of its clinical efficacy and tolerability data and concluded that escitalopram did not have notable efficacy or tolerability advantages over other marketed antidepressants (Food and Drug Administration, 2002). However, there have been many studies conducted since that time. This qualitative review of the literature sought to examine the more recent clinical literature and assess whether there is evidence of a clinical advantage for escitalopram over either citalopram or other frequently used antidepressants.

## Methods

Searches were conducted using PubMed and EMBASE. With PubMed, several independent searches were performed. The keyword ‘escitalopram’ was used in combination with ‘placebo’, ‘citalopram’, ‘sertraline’, ‘paroxetine’, ‘fluoxetine’, ‘duloxetine’ and ‘venlafaxine’ in separate searches. All searches were limited to trials in adults, clinical trial and English language. Searches were limited to the title/abstract fields. The four searches were then cross-referenced to avoid duplication. For the EMBASE search, the keyword ‘escitalopram’ was used alone and the search limited to English language and clinical trials. All retrieved titles were then cross-referenced with the searches from PubMed to avoid duplication. No date limits were applied to any of the searches; however, the searches were completed in January 2009 and do not, therefore, include studies published after this date. Published congress abstracts or posters were not included. Abstracts of the retrieved studies were reviewed independently by both authors for inclusion in the analysis and any discrepancies resolved by discussion. Of the retrieved studies, only those pertaining to depression or MDD were selected. All articles were reviewed. Clinical trials were limited to randomized controlled trials (RCTs). Open-label, community-based or naturalistic studies were not included in the analysis; however, findings of the analysis are discussed in the context of the results from these studies and pharmaco-economic studies. Data were extracted for trial design, participant characteristics and outcomes according to the criteria for RCTs as detailed in the Consolidated Standards of Reporting Trials (CONSORT group, 2009) and the Critical Appraisal Skills Programme (National Health Service, 2009).

## Results

The search identified over 250 citations, of which 21 RCTs and 18 pooled studies or meta-analyses in MDD were identified as being suitable for inclusion. The design and outcomes of the RCTs are summarized in Table 1 (available online). A summary of the findings from the published meta-analyses are shown in Table 2 (available online).

### Assessment of escitalopram efficacy

#### Studies comparing escitalopram with placebo

A large number of studies have compared escitalopram with placebo, either with or without an active comparator (i.e. citalopram, duloxetine or fluoxetine), and their results largely show a benefit of treatment with escitalopram (Bose and Gandhi, 2008; Burke et al., 2002; Kasper et al., 2005; Lepola et al., 2003; Nierenberg et al., 2007; Rapaport et al., 2004; Wade et al., 2002). In five out of seven studies, escitalopram was significantly better than placebo with respect to all measures of depression (Montgomery-Åsberg depression rating scale (MADRS), Hamilton rating scale for depression (HAM-D) and clinical global impression (CGI) scales) (Burke et al., 2002; Lepola et al., 2003; Nierenberg et al., 2007; Rapaport et al*.*, 2004; Wade et al., 2002).

Meta-analyses and pooled analyses are important complementary strategies that can be used to compare different therapeutic options (Thase, 2002). The analyses of Wade and Friis Andersen (2006), Llorca et al. (2005), Lepola et al. (2004) and Gorman et al. (2002) all showed a statistically significant improvement in MADRS score with escitalopram compared with placebo after the first week of treatment. In patients who responded after two weeks of treatment, and continued treatment for the entire eight weeks, 63% were in remission at the end of the study (Wade and Friis Andersen, 2006). Similarly, an analysis by Clayton et al. (2006) revealed that escitalopram was superior to placebo (and bupropion). A further two analyses explored the impact of disease severity on the efficacy of escitalopram compared with placebo; findings were contradictory, with one analysis reporting a greater difference between escitalopram and placebo in patients who were severely depressed at baseline (Lam and Andersen, 2006), and the other demonstrating superiority of escitalopram (10 mg) over placebo in moderately depressed patients, and superiority of escitalopram (20 mg) over placebo in severely depressed patients (Bech et al., 2006). More recently, the meta-analysis by Kennedy et al. (2009) showed a significant between treatment effect of 2.3 MADRS points, representing a significant clinical benefit favouring escitalopram versus placebo in MDD.

In the analysis by Svensson and Mansfield (2004), which reviewed the efficacy of escitalopram compared with both placebo and citalopram by analysing both published and unpublished data, the authors challenge the efficacy findings with escitalopram, citing potential methodological flaws in the studies that could account for the differences between escitalopram and both placebo and citalopram. It is worth noting that this early analysis included only a small proportion of the studies comparing the efficacy of escitalopram with that of citalopram that have been published to date. Subsequent to the publication of the analysis by Svensson and Mansfield (2004), a total of four clinical studies (Colonna et al., 2005; Lepola et al., 2003; Moore et al., 2005; Yevtushenko et al., 2007) and five pooled analyses (Einarson, 2004; Lader et al., 2005; Lam and Andersen, 2006; Lepola et al., 2004; Llorca et al., 2005) were published, and these provide further evidence to support the efficacy findings reported for escitalopram compared with citalopram.

#### Studies comparing escitalopram with citalopram

A total of five randomized, double-blind studies have directly compared the efficacy of escitalopram (10–20 mg) with that of citalopram (10–40 mg) in more than 1700 patients. The mean change in MADRS score was significantly greater in patients receiving escitalopram than citalopram at both six (Yevtushenko et al., 2007) and eight weeks (Burke et al., 2002; Colonna et al., 2005; Lepola et al., 2003; Moore et al., 2005). At 24 weeks, however, there was no significant difference in efficacy between citalopram and escitalopram (Colonna et al., 2005). In the two placebo-controlled, randomized, double-blind studies in which citalopram was used as an active comparator, analysis of time to separation from placebo showed that escitalopram (10–20 mg) resulted in a statistically significant separation from placebo on all rating scales during weeks 1–2, compared with weeks 6–8 for citalopram (20–40 mg) (Burke et al., 2002; Lepola et al., 2003). This suggests that treatment with escitalopram is associated with earlier relief of depressive symptoms compared with citalopram (Kasper et al., 2006b), which is in keeping with findings from preclinical studies (Sanchez, 2006; Sanchez et al., 2004). Only a single study reported an increased prevalence of adverse events with citalopram compared with escitalopram (Yevtushenko et al., 2007); in all other studies, both treatments were found to be equally safe and well tolerated (Burke et al., 2002; Colonna et al., 2005; Lepola et al., 2003; Moore et al., 2005).

Furthermore, three pooled analyses have compared the efficacy of escitalopram with that of citalopram (Gorman et al., 2002; Lepola et al., 2004; Llorca et al., 2005). In these three studies, escitalopram was superior to citalopram with respect to MADRS score (Gorman et al., 2002; Lepola et al., 2004; Llorca et al., 2005). In the study by Lepola et al. (2004), for example, the mean decrease from baseline in MADRS total score in the results of the pooled studies was greater after eight weeks of treatment in escitalopram-treated patients than in those treated with citalopram, even in a subpopulation of severely ill patients (i.e. those with MADRS score of ≥30). Two early pooled analyses of the initial registration trials showed a faster onset and superior efficacy of escitalopram compared with citalopram (Gorman et al., 2002; Lepola et al., 2004). Data on speed of onset need to be viewed cautiously because statistical separation is partly dependent on subject numbers (Taylor et al., 2006b).

Recently, Kennedy et al. (2009) demonstrated a significant treatment difference in terms of MADRS, remission rates and responder rates favouring escitalopram versus SSRIs in people with MDD, and the main source of these differences were due to differences between escitalopram versus citalopram. Similar findings were obtained in an analysis of patients with severe depression at baseline.

Positive effects of escitalopram on measures of sleep have been shown in a study by Lader and colleagues in comparison with citalopram (Lader et al., 2005). Patients with MDD associated with sleep problems, defined as MADRS item 4 score ≥4, were treated with escitalopram, citalopram or placebo. Patients treated with escitalopram showed improvement in mean MADRS item 4 score at weeks 4, 6 and 8 compared with patients treated with both placebo (*p* < 0.05) and citalopram (*p* < 0.01). Furthermore, few patients (<6%) reported sleep-related, treatment-emergent adverse events, such as daytime somnolence and/or insomnia (Lader et al., 2005).

#### Studies comparing escitalopram with other selective serotonin reuptake inhibitors

The efficacy of escitalopram in MDD has been compared with that of paroxetine in two randomized, double-blind studies. Escitalopram elicited a statistically significant improvement in MADRS, HAM-D and CGI-severity (CGI-S) scores following 8 weeks of treatment (Boulenger et al., 2006) and, in a subgroup of severely depressed patients, significant improvement in MADRS score was detected at 27 weeks (Baldwin et al., 2006). Furthermore, not only was there an increase in the rate of treatment discontinuations owing to adverse events in paroxetine compared with escitalopram-treated patients (Baldwin et al., 2006; Boulenger et al., 2006), but significantly more patients in the paroxetine group withdrew from the study owing to lack of efficacy (Baldwin et al., 2006).

Only two randomized, double-blind studies compared the efficacy of escitalopram with that of fluoxetine and these studies have reported conflicting results. The study by Mao et al. (2008) in Chinese patients showed no significant difference between the two treatments at 8 weeks, although escitalopram was superior with respect to both the ‘depressed mood’ and ‘work and interests’ items of the HAM-D scale. The second study, by Kasper et al. (2005) in elderly patients, suggested that although fluoxetine was significantly less efficacious than both escitalopram and placebo, escitalopram in turn was not superior to placebo. The adverse-event profile of the two antidepressants was similar (Mao et al., 2008), but there were a greater number of treatment discontinuations with fluoxetine compared with escitalopram owing to either adverse events or lack of efficacy (Kasper et al., 2005).

A single study has explored the comparative efficacy of escitalopram and sertraline and found no differences between agents (Ventura et al., 2007). However, in this study a fixed dose of escitalopram of 10 mg/day, which is the lowest therapeutic dose, was compared with sertraline flexibly dosed across its entire recommended dose range of 50–200 mg/day (mean dose at week 8: 144 mg/day). The similarity in the efficacy of these two antidepressants reported in this clinical study may not, therefore, translate into clinical practice.

#### Studies comparing escitalopram with serotonin-noradrenaline reuptake inhibitors

The efficacy of escitalopram has been directly compared with that of venlafaxine XR in two randomized, double-blind studies, in a total of 484 patients, and found to be very similar (Bielski et al., 2004; Montgomery et al., 2004), but with some advantage of escitalopram in patients with severe MDD (Montgomery et al., 2006). Three randomized, double-blind studies have compared the efficacy of escitalopram and duloxetine (Khan et al., 2007; Nierenberg et al., 2007; Pigott et al., 2007; Wade et al., 2007). Two of these revealed that patients treated with escitalopram had greater improvements in MADRS score (Khan et al., 2007; Wade et al., 2007): one study showed a statistically significantly greater efficacy for escitalopram compared with duloxetine at eight weeks (Khan et al., 2007); the other demonstrated that the mean change from baseline to week 8 in MADRS score was significantly greater in escitalopram- versus duloxetine-treated patients (Wade et al., 2007). Another study, designed to establish non-inferiority, demonstrated that duloxetine had as fast an onset of efficacy as escitalopram and was associated with similar efficacy over the 8 weeks of the study: 42.6% and 35.2% of patients had a 20% sustained reduction in HAM-D Maier subscale score with duloxetine and escitalopram, respectively (*p* = 0.097). Both treatments were better than placebo (*p* < 0.001 for duloxetine versus placebo and *p* = 0.008 for escitalopram versus placebo) (Nierenberg et al., 2007). However, in a six-month extension of this study, there was no significant difference in efficacy between escitalopram and duloxetine, although escitalopram did have a significantly improved effect on sleep (Pigott et al., 2007). In a pooled analysis of two clinical studies by Lam and Wade, the incidence of nausea, insomnia, dizziness and vomiting was statistically significantly higher in patients treated with duloxetine compared with those treated with escitalopram; however, the incidence of upper respiratory tract infection was statistically significantly higher in patients treated with escitalopram (Lam et al., 2008). Overall, the authors concluded that escitalopram demonstrated superior tolerability, as evidenced by lower overall withdrawal rates as a result of adverse events (Lam et al., 2008). These findings mirror those from individual studies comparing escitalopram and duloxetine (Khan et al., 2007; Nierenberg et al., 2007; Pigott et al., 2007; Wade et al., 2007).

#### Meta-analysis of studies comparing escitalopram, selective serotonin reuptake inhibitors and serotonin-noradrenaline reuptake inhibitors

**Figure 1.**
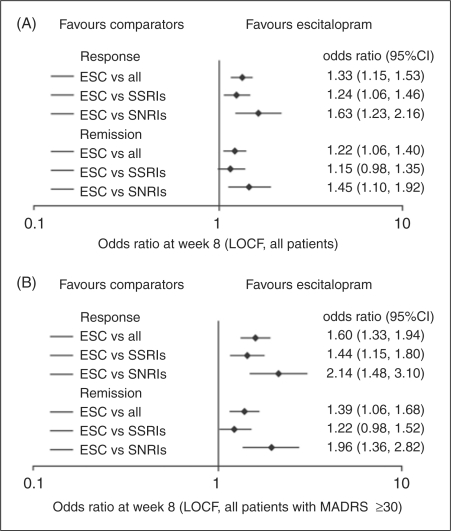
Response and remission rates^a^ following treatment at end of study (A) for all patients and (B) for severely depressed patients (permission pending from Kennedy et al., 2009). ^a^Response (defined as a ≥50% reduction in baseline MADRS total score; LOCF) and remission (defined as MADRS total score ≤12; LOCF) rates. Data are odds ratios with 95% CIs. CI: confidence interval; ESC: escitalopram; LOCF: last observation carried forward; MADRS: Montgomery-Asberg depression rating scale; SNRI: selective norepinephrine reuptake inhibitor; SSRI: selective serotonin reuptake inhibitor.

In addition to the numerous head-to-head trials of escitalopram and individual SSRIs and serotonin-norepinephrine reuptake inhibitors (SNRIs), several meta-analyses were identified that examined the relationships between antidepressants in terms of their efficacy and tolerability. One meta-analysis revealed that escitalopram had superior efficacy compared with other antidepressants (e.g. citalopram, fluoxetine, paroxetine, sertraline and venlafaxine XR), as assessed by MADRS on a series of endpoint comparisons involving change in efficacy scores from baseline, and response and remission rates (Kennedy et al., 2006). This meta-analysis included 10 clinical trials and was designed to detect an overall difference between escitalopram and other antidepressants as a group. It did not draw firm conclusions on the comparative efficacy of escitalopram and any one of the individual antidepressants listed, as the number of studies directly comparing escitalopram with these individual antidepressants was low. Another meta-analysis by Kasper et al. (2006b) reported similar findings: the mean change in MADRS score was found to be significantly higher in patients treated with escitalopram after one week of treatment, and this benefit persisted for the duration of time that assessments were made. Interestingly, the CGI-S scores also revealed that escitalopram was associated with a faster onset of action than other antidepressants. An earlier meta-analysis confirmed the superiority of escitalopram versus citalopram with respect to both response and remission rates, but found no difference between escitalopram and venlafaxine XR (Einarson, 2004). This is perhaps not surprising given the small number of patients who received venlafaxine XR in the single study included in this analysis. Meanwhile, a more recent meta-analysis by Kennedy et al. (2009), which included 16 studies of escitalopram of eight weeks’ duration or longer in the treatment of MDD, revealed that escitalopram was associated with more favourable MADRS scores and remission and responder rates compared with placebo, SSRIs and SNRIs (Figure 1), and the results were comparable when the analyses were restricted to patients with severe depression (baseline MADRS ≥30), although the benefit of escitalopram versus SSRIs was largely due to differences between escitalopram versus citalopram.

The recent meta-analysis by Cipriani et al. (2009) concluded that there were clinically important differences between antidepressants in terms of efficacy (response rate) and acceptability (patient discontinuation); the conclusions were in favour of escitalopram and sertraline. This independently funded meta-analysis systematically reviewed 117 RCTs and evaluated 12 commonly prescribed antidepressants, including citalopram, escitalopram, duloxetine, fluoxetine, paroxetine, sertraline and venlafaxine, in 25,928 patients with unipolar MDD. The methodology used differed from other meta-analyses, since it incorporated a matrix design to allow multiple treatment comparisons. The meta-analysis did not include outcomes such as side-effects, toxic effects, discontinuation symptoms and social functioning. Mirtazapine, escitalopram, venlafaxine and sertraline were more efficacious than duloxetine, fluoxetine, fluvoxamine, paroxetine and reboxetine. Escitalopram, sertraline, citalopram and bupropion were judged to be more acceptable than the other antidepressants evaluated. Thus, escitalopram and sertraline were established as treatments of choice in depression.

### Assessment of escitalopram tolerability and safety

In terms of safety and tolerability, data for escitalopram are available from over 4000 patients in double-blind treatment, representing approximately 1000 patient-years of exposure (Baldwin et al., 2007). Overall, the adverse-event profile detailed in published studies and meta-analyses is consistent with the known serotonergic properties of escitalopram. A meta-analysis of safety data from 24 double-blind, active-comparator or placebo-controlled, randomized studies with escitalopram was recently published (Baldwin et al., 2007). The most common adverse events (incidence ≥5%) reported during the first 8 weeks of treatment with escitalopram, which were significantly more frequent than in the placebo group, were nausea, insomnia, fatigue, diarrhoea, dizziness, dry mouth, somnolence and ejaculation failure (Table 3, available online). The majority of these events were mild to moderate in severity (Baldwin et al., 2007).

From weeks 8 to 24, only nasopharyngitis (escitalopram 3.2% vs comparator 2.9%) and headache (escitalopram 2.5% vs comparator 2.6%) had an incidence ≥2% (Baldwin et al., 2007). Importantly, particularly for a treatment used long term in clinical practice, in the heterogeneous patient samples studied, escitalopram was both safe and well tolerated (Anders et al., 2008; Kasper et al., 2006a; Wade et al., 2006). No new types of adverse events were seen in long-term treatment compared with acute treatment (Baldwin et al., 2007; Kasper et al., 2006a; Wade et al., 2006). Furthermore, weight gain, which is frequently cited as reason for non-adherence to long-term SSRI treatment, was not seen after 24 weeks of treatment with escitalopram (Baldwin et al., 2007).

In the same meta-analysis, the safety profile of escitalopram was also compared with other antidepressants, including citalopram, fluoxetine, paroxetine, sertraline and venlafaxine XR. The withdrawal rate at 8 weeks owing to adverse events was significantly lower for escitalopram compared with both paroxetine and venlafaxine XR, and escitalopram resulted in fewer discontinuation signs and symptoms than paroxetine (Baldwin et al., 2007). The latter finding is mirrored by a study that utilized telephone calls reporting symptoms of antidepressant withdrawal received by the UK national medication helpline between October 1997 and March 2005. Of the total of 1753 calls, 39.4% related to paroxetine, whereas 0.9% related to escitalopram. Furthermore, the numbers of calls relating to symptoms of withdrawal were also high with both venlafaxine XR (14.4%) and citalopram (8.0%) compared with escitalopram. However, it is worthwhile noting that the number of calls relating to symptoms of withdrawal is also dependent on the number of prescriptions written, and the same study reported that a much higher number of prescriptions was dispensed for paroxetine (24.3 million), venlafaxine (12.2 million) and citalopram (19.3 million) compared with escitalopram (1.75 million) (Taylor et al., 2006b).

Three randomized, double-blind studies have assessed the safety of escitalopram compared with that of duloxetine, which were not included in the analysis by Baldwin et al. (2007). Duloxetine was associated with an increased incidence of adverse events, particularly insomnia and constipation (Nierenberg et al., 2007; Wade et al., 2007), as well as significant increases in the incidence of nausea, dry mouth, yawning and vomiting (all *p* < 0.05) (Nierenberg et al., 2007), and in two out of the three studies, these adverse events accounted for an increase in the rate of discontinuation in patients treated with duloxetine compared with those treated with escitalopram (Khan et al., 2007; Wade et al., 2007). Although, in one study, escitalopram was associated with a higher incidence of treatment-emergent sexual dysfunction at 4 weeks when compared with duloxetine, there was no significant difference at 12 weeks and no difference in the rates of discontinuations due to sexual dysfunction (Clayton et al., 2007).

## Discussion

### Clinical benefits of escitalopram compared with other antidepressants

Over the last five years, a large body of evidence has accumulated establishing the clinical efficacy and safety of the *S*-enantiomer, escitalopram compared with other antidepressants (see summary points below). These findings provide further evidence to support the efficacy findings reported for escitalopram compared with citalopram, which had previously been disputed by Svensson and Mansfield (2004). It should also be noted that during peer-review of our manuscript, a post-hoc pooled analysis of data from two 6-month RCTs was published, which concluded that escitalopram is a good therapeutic option for the long-term treatment of MDD (Kasper et al., 2009). In addition, an integrative analysis of four double-blind, randomized, clinical trials, that was published during peer review of our manuscript, concluded that escitalopram is at least as effective as the SNRIs (venlafaxine XR and duloxetine), even in severe depression, and was better tolerated (Kornstein et al., 2009)

Although only a small number of studies have directly compared escitalopram with paroxetine, fluoxetine and sertraline, these SSRIs have been included in several meta-analyses, which have evaluated the outcome of multiple clinical trials assessing the efficacy and tolerability of antidepressants. The most recent, and perhaps most compelling meta-analysis, by Cipirani et al. (2009), concluded that escitalopram and sertraline might be the best choice of antidepressant when starting treatment of patients with moderate to severe MDD, since they offer the best ratio of efficacy and acceptability. It is noteworthy that escitalopram and citalopram were compared separately in this meta-analysis (Cipriani et al., 2009).

A number of meta-analyses suggest that the SNRI venlafaxine XR is associated with greater remission or response rates in patients with MDD compared with SSRIs (Nemeroff et al., 2008; Papakostas et al., 2007; Smith et al., 2002; Thase, 2008; Thase et al., 2005). These results have prompted some authors to debate whether dual-action antidepressant drugs that combine both a serotonergic and noradrenergic mechanism of action are more effective in patients with MDD than the SSRIs (Papakostas et al., 2007). The evaluation of the efficacy of escitalopram in comparison with the SNRIs venlafaxine XR and duloxetine may have been limited by the small number of studies directly comparing these agents; however, these studies were performed in a large number of patients (>1500 patients) and, thus, were sufficiently powered to identify important clinical differences between the treatments (Cipriani et al., 2009).

Summary points for efficacy:

- efficacy advantage for escitalopram versus citalopram has been demonstrated in five randomized studies (Burke et al., 2002; Colonna et al., 2005; Lepola et al., 2003; Moore et al., 2005; Yevtushenko et al., 2007) and two pooled analyses of clinical trials (Gorman et al., 2002; Lepola et al., 2004);
- escitalopram may have an efficacy advantage over paroxetine, in terms of significant improvements in MADRS, HAM-D and CGI-S scores and fewer discontinuations owing to lack of efficacy (Baldwin et al., 2006; Boulenger et al., 2006);
- the efficacy of escitalopram compared with fluoxetine (Mao et al., 2008; Kasper et al., 2005) and sertraline (Ventura et al., 2007) requires further evaluation;
- escitalopram and sertraline might be the best choice of antidepressant when starting treatment of patients with moderate to severe MDD (Cipriani et al., 2009);
- the SNRI venlafaxine XR may be associated with greater remission or response rates in patients with MDD compared with SSRIs (Papakostas et al., 2007; Smith et al., 2002; Nemeroff et al., 2008; Thase, 2008 Thase et al., 2005; Burk et al., 2002);
- escitalopram has comparable efficacy to venlafaxine XR, with a possible slight advantage for escitalopram in patients with severe MDD (Bielski et al., 2004; Montgomery et al., 2004)

Regarding safety and tolerability, escitalopram offers tolerability advantages over duloxetine, paroxetine and venlafaxine XR (see summary points below) and may be preferable to those antidepressants in some patients with MDD.

Summary points for safety:

- superior tolerability for escitalopram versus duloxetine, particularly for nausea, insomnia, dizziness and vomiting has been demonstrated in three randomized studies (Khan et al., 2007; Nierenberg et al., 2009; Wade et al., 2007) and a pooled analysis of two clinical trials (Lam et al., 2008)
- escitalopram offers tolerability advantages over paroxetine (Baldwin et al., 2006; Boulenger et al., 2006) and venlafaxine XR (Bielski et al., 2004; Montgomery et al., 2004, 2006);
- escitalopram is less likely to cause discontinuation symptoms than several other antidepressants (Baldwin et al., 2006; Bielski et al., 2004; Boulenger et al., 2006; Kasper et al., 2005; Khan et al., 2007; Montgomery et al., 2004, 2006; Taylor et al., 2006a; Wade et al., 2007);
- no evidence of emergent risk of suicide with escitalopram (Baldwin et al., 2007; Pedersen, 2005);
- incidence of sexual dysfunction-related adverse events similar to citalopram, but lower than venlafaxine XR and paroxetine (Baldwin et al., 2007).

### Why might escitalopram confer clinical advantages to citalopram?

Careful consideration of a drug’s stereochemistry is becoming increasingly important when developing new antidepressants to provide greater insight into the pharmacological subtleties of the agents being prescribed in clinical practice (Baumann et al., 2002). It can be postulated that the observed clinical benefits of escitalopram compared with citalopram are conferred by the pharmacological and pharmacodynamic properties of the constituent enantiomers. These properties are summarized below.

In addition to overall clinical efficacy in terms of symptom improvement, another potential clinical advantage that escitalopram may confer compared with citalopram is earlier onset of action (Kasper et al., 2006b). Increasing attention has been focused on the requirement for antidepressant medications to have a rapid onset of effect (Blier, 2003; Rosenbaum, 2001; Taylor et al., 2006b), since this can translate into clinical benefit in terms of symptom control and treatment continuation (Kasper et al., 2006b). Initial clinical results (Kasper et al., 2006b), which were identified in the literature search (Table 1, available online), suggested that escitalopram had a faster onset of action compared with citalopram (see summary points below). It has been suggested that the reduction in serotonergic activity as a result of autoreceptor activation is the rate-limiting step in the clinical response to antidepressants. Therefore, more rapid desensitization of the receptors, as observed with escitalopram, could lead to a faster onset of action (El Mansari et al., 2005).

Summary points for escitalopram versus citalopram:

- escitalopram (*S*-enantiomer) has improved pharmacological potency as a serotonin reuptake inhibitor in vitro versus the *R*-enantiomer; the *S*-enantiomer has the benefit of allosterically modulating the SERT; (Hyttel et al., 1992)
- the *R*-enantiomer is metabolized more slowly than the *S*-enantiomer (Sidhu et al., 1997), resulting in approximately two-fold higher plasma concentration of the *R*-enantiomer (Overo, 1982; Tanum et al., 2003);
- *R*-citalopram may have a functional antagonistic effect on escitalopram (Cremers and Westerink, 2003; El Mansari et al., 2005; Lucki and Brown, 2003; Mørk et al., 2003;) with significantly higher occupancy 6 h post-dose with escitalopram (10 mg/day: 82%) compared with citalopram (20 mg/day: 64%) (Klein et al., 2007; Meyer et al., 2004);
- *R*-citalopram should not be considered an inactive enantiomer: it may have a deleterious influence on the activity of escitalopram through functional antagonism (El Mansari et al., 2007);
- escitalopram may have a faster onset of action than citalopram (Sanchez, 2003; Sanchez et al., 2003a, b) as a result of more rapid desensitization of 5-hydroxytryptamine_1A_ subtype autoreceptors, leading to more rapid disinhibition of the serotonergic neurones, and enhanced release of serotonin (Blier and Bouchard, 1994).

### Translating clinical efficacy into ‘real-life’ benefit

The outcomes of naturalistic studies can provide insight into the use of escitalopram in the wide range of patients with MDD seen in clinical practice. While evidence-based medicine is the main platform upon which treatment efficacy is evaluated, it is important that the findings from RCTs translate into real, everyday clinical practice. The patient populations of RCTs are typically homogeneous as they are determined based on the stringent inclusion and exclusion criteria required for regulatory approval of a specific indication; therefore, for a treatment to be clinically relevant, the results from RCTs must apply to the broader population of patients treated in psychiatric and primary care practices. However, although studies conducted under ‘real-life’ conditions provide valuable insight into the efficacy of the drug in a population that is representative of outpatients suffering from depression, the findings from such studies cannot be included in meta-analyses that determine the overall treatment effect. A number of such naturalistic studies have been conducted with escitalopram and their findings are consistent with those reported in RCTs (Lancon et al., 2006; Moller et al., 2007; Rush and Bose, 2005). In these studies, 8 weeks of treatment with escitalopram was associated with improvements in measures of depression, including MADRS score and CGI-improvement score (Moller et al., 2007; Rush and Bose, 2005). These findings were corroborated by a large-scale, uncontrolled open-label study of Moller et al., in which over 11,500 patients were treated and 83% showed much or very much improved CGI-I (Moller et al., 2007).

The long-term efficacy of escitalopram, in terms of improved symptom severity, has been demonstrated in a number of six- or 12-month open-label studies (Anders et al., 2008; Kasper et al., 2006a; Wade et al., 2006), in which the majority of patients (72% to 86%) achieved remission (MADRS ≤12). It would, therefore, appear that the mechanistic advantages of escitalopram over citalopram, in terms of increased potency (Hyttel et al., 1992) and its known effect on the SERT through an affinity-modulating allosteric site (Chen et al., 2005), translate into clinical benefits in patients with MDD, as demonstrated in the pooled analysis by Llorca et al. (2005), which showed significantly greater mean change from baseline in MADRS with escitalopram versus citalopram (56% vs 41%**,** respectively; *p* = 0.007).

In addition to the requirement for efficacy and safety, it is pertinent that an antidepressant agent is cost-effective. In 2007, the cost of treating depression, including both direct and indirect costs, was estimated at €118 billion per year in Europe (Lam and Annemans, 2007). Several pharmaco-economic studies have suggested that escitalopram may be more cost-effective than citalopram (Croom and Plosker, 2003; Demyttenaere et al., 2005; Fantino et al., 2007; François et al., 2003; Hemels et al., 2004; Sorensen et al., 2007; Wade et al., 2005a, b; Wu et al., 2008) and duloxetine (Wade et al., 2008) and at least as cost-effective as venlafaxine XR (Croom and Plosker, 2004; Demyttenaere et al., 2005; Fernandez et al., 2005; Kulp et al., 2005; Llorca and Fernandez, 2007; Sorensen et al., 2007). These results suggest that the improved efficacy of escitalopram, both in clinical trials and in ‘real life', translate into improved cost-effectiveness, thereby relieving some of the burden of depression on healthcare authorities and this is supported by Cipriani et al (Cipriani et al., 2009). However, it should be noted that the cost-effective analyses were conducted when venlafaxine XR was under patent and this should be considered when interpreting these data.

### Limitations of this literature review

It is important to acknowledge a number of potential limitations of this literature review article, which are common to all review articles of this nature. These include publication bias, i.e. the selective publication of trials with positive results, and differences in study design, including dosing regimens, sample sizes and inclusion criteria (Davis et al., 1995; Davey Smith and Egger, 1998; Egger and Smith, 1998).

An important consideration when interpreting these findings is the issue of dose comparability. Ideally, individual studies included in any review, qualitative or quantitative, should involve dosing across the full regulatory-approved dose range for each treatment group. If this is not the case, in order to ensure fair comparability between the two treatment groups, the study should assess the efficacy of minimal doses of drug A with minimal doses of drug B, and high doses should be compared with high doses (Lieberman et al., 2005). The dose of escitalopram used across the studies included in this review ranged from 10 to 20 mg, which is the dose range approved in Europe. All of the included studies comparing escitalopram and citalopram involved comparable doses. However, it should be pointed out that the majority of these comparative studies used fixed doses of escitalopram and citalopram and it is possible that these fixed-dose studies simply used more effective doses of escitalopram than citalopram. Some of the comparative studies with other antidepressants cannot be considered to be dose comparable and this should, therefore, be considered when interpreting their findings; for example, in the study by Montgomery and colleagues, 10–20 mg of escitalopram was compared with 75–150 mg of venlafaxine XR (Montgomery et al., 2004).

In addition, many of the studies included here involved relatively small samples of patients, which may limit interpretation owing to insufficient power to detect reliably the differences between two effective treatments (Thase, 1999, 2002). However, the combining of data in meta-analyses helps to overcome this potential limitation.

Finally, although changes in scores on clinical scales such as the MADRS and HAM-D, are well-established measures of clinical efficacy, antidepressants can also be assessed using validated surrogate outcomes, which may be better measures of ‘real-life’ efficacy, such as remission rates, quality of life, patient-reported outcomes and productivity (Lam and Annemans, 2007); for example, the recent study by Demyttenaere et al. (2008), showed that patients treated with escitalopram reported a statistically and clinically significant improvement in quality of life enjoyment and satisfaction using data from eight randomized, eight-week, clinical trials. This review did not assess efficacy in terms of these patient-centred outcomes; however, a number of observational or naturalistic studies were included, and these can be assumed to better capture treatment efficacy in the ‘real-life’ clinical setting.

## Conclusions

In the recent past it has been thought that there is very little difference in both efficacy and safety between antidepressants currently available. However, there is evidence suggesting that the mechanistic advantages of escitalopram over the *R*-enantiomer and racemic citalopram translate into clinical efficacy benefits, both in the context of clinical studies and the ‘real-life’ setting. In addition, there is evidence that the onset of treatment response is earlier with the *S*-enantiomer alone.

Evidence for an efficacy advantage over other SSRIs and SNRIs is less robust, but escitalopram does appear to offer some tolerability advantages over several other antidepressants, particularly the SNRIs, venlafaxine XR and duloxetine. Escitalopram is, therefore, associated with a favourable benefit-risk ratio compared with some other antidepressant agents. These advantages, seem to be associated with the drug's unique chemistry and pharmacological actions.
